# Estimating scientific coherence using population-level indicators and research production data: a longitudinal analytical proof-of-concept study

**DOI:** 10.3389/frma.2026.1817595

**Published:** 2026-06-29

**Authors:** David A. Hernandez-Paez, Fabriccio J. Visconti-Lopez, Ivan David Lozada-Martınez

**Affiliations:** 1 Center for Meta-Research and Scientometrics in Biomedical Sciences, Barranquilla, Colombia; 2 Universidad Cientdel Sur, Lima, Peru; 3 Clínica Colsanitas S.A., Clínica Iberoamérica, Barranquilla, Colombia; 4 Biomedical Scientometrics and Evidence-Based Research Unit, Department of Health Sciences, Universidad de la Costa, Barranquilla, Colombia

**Keywords:** biomedical research, epidemiologic methods, health status indicators, meta-research, proof-of-concept study

## Abstract

The rapid expansion of scientific research is frequently assumed to translate into improvements in population health and development outcomes, yet methods to empirically evaluate this alignment remain limited. Existing bibliometric and impact-based approaches describe scientific activity but rarely examine its longitudinal relationship with population-level indicators. We introduce the concepts of scientific coherence and development coherence, referring to measurable associations between research production, population indicators, and structural determinants over time. To operationalize these concepts, we propose the Data-driven Analysis and Inference of Longitudinal population indicators and research production (DAIL) framework, a three-step analytical pipeline integrating regression models, hierarchical mixed-effects analyses, and moderator screening. A proof-of-concept application illustrates how longitudinal associations between research production and global indicators can be quantified using widely available data. While our approach quantifies these longitudinal patterns, we explicitly acknowledge the inherent potential for reverse causality, recognizing that favorable socioeconomic conditions and structural development may act as prerequisites for sustaining a functioning academic research infrastructure, rather than acting strictly as outcomes of expanded research. This framework provides a methodological basis for studying the co-evolution and alignment between scientific activity and population dynamics in epidemiology.

## Introduction

In recent decades, the volume of scientific research has increased substantially across most fields of health and biomedical sciences (González-Márquez et al., 2024; Bornmann and Mutz, 2015). Large-scale investments in research and innovation are frequently justified on the premise that expanding scientific knowledge ultimately contributes to improvements in population health, social conditions, or development indicators (Terama et al., 2016; Thonon et al., 2015). However, despite this implicit

assumption, there is still limited methodological capacity to empirically evaluate whether increases in research production are temporally and structurally aligned with changes in population-level outcomes (Terama et al., 2016; Thonon et al., 2015).

Most existing approaches to assess the broader alignment of research rely on bibliometric indicators, citation-based metrics, or evaluations of knowledge translation processes (Moutinho, 2024). Although these approaches provide valuable information about the dissemination and visibility of scientific output, they rarely examine whether scientific activity is associated with measurable changes in population indicators over time. As a result, an important dimension of research evaluation remains insufficiently explored: the extent to which scientific production and population-level dynamics evolve in a coherent manner (Subbiah, 2023).

In epidemiology and population health research, longitudinal and ecological analyses are routinely used to study associations between exposures and outcomes across populations and over time (Riggs et al., 2024). Similar analytical principles could, in theory, be applied to examine the relationships between research production and population indicators (Picón-Jaimes et al., 2025; Lozada-Martinez et al., 2025). However, there is currently no widely adopted framework designed specifically to estimate such relationships in a systematic and reproducible way.

In this context, we introduce the concept of scientific coherence, defined as the extent to which variations in research production are associated with changes in population-level indicators in a temporally and structurally consistent manner. Under this perspective, scientific coherence does not imply causality but rather refers to measurable patterns of association that may reflect alignment, lagged relationships, or divergence between research activity and population dynamics.

A related concept is development coherence, which refers to the extent to which changes in structural or socioeconomic conditions are associated with variations in research production over time. While scientific coherence focuses on the relationship from research to population indicators, development coherence examines the inverse direction, acknowledging that scientific production itself is influenced by broader demographic, economic, and institutional contexts.

Understanding these relationships is important for epidemiology and public health for several reasons. First, population-level indicators are increasingly used to monitor progress toward health and development goals, yet little is known about how scientific activity relates to these trajectories (Samet and Hussein, 2024). Second, evaluating the alignment between research production and population needs may help identify areas where scientific efforts are either insufficient, reactive, or poorly translated into measurable outcomes (Terama et al., 2016; Thonon et al., 2015). Third, methodological tools capable of integrating longitudinal research production data with population indicators may contribute to a more evidence-based approach to research prioritization and policy planning (Picón-Jaimes et al., 2025; Lozada-Martinez et al., 2025).

In this study, we propose a longitudinal analytical framework to estimate scientific coherence between research production and population-level indicators. Using population-level datasets and panel modeling approaches, we illustrate how such relationships can be quantified and explored across time and population strata through a proof-of-concept analysis.

## What is scientific coherence?

When research is produced, three scenarios can reasonably be expected. As the volume of research increases, the indicators associated with that research may (1) improve, (2) deteriorate, or (3) remain unaffected. In this paper, we introduce the concept of scientific coherence, which corresponds to the first scenario: the expectation that increased research production co-evolves positively with the outcomes to which it is related.

For example, if disease A is studied more extensively than disease B, one would expect that outcomes associated with disease A, such as prevalence, mortality, and the accessibility and availability of therapies, are more favorable than those of disease B. This expectation is not limited to diseases alone, but extends to any research domain and its associated indicators.

Scientific coherence, therefore, represents an ideal that should be embedded within the global research agenda. Under this ideal, the majority of research efforts would address genuine needs, whether by identifying problems, proposing solutions, or testing the validity of strategies designed to address them (Robinson et al., 2021; Lund et al., 2021a,b). As can be inferred, this ideal is sometimes achieved, corresponding to the first scenario, but at other times it is not, giving rise to the remaining two scenarios. Consequently, a framework capable of estimating the alignment of global research with population dynamics becomes necessary.

## What is development coherence?

It is also true that scientific production is influenced by structural factors. These structural factors may include a range of socioeconomic domains which, in the contemporary world, can be quantified through a wide array of indicators. Among them are gross domestic product per capita, political corruption indices, average years of schooling, poverty indices, the Gini index, and many others. Enumerating all of these would not only exceed the reasonable length of the present manuscript but is also unnecessary, as such indicators are freely available as longitudinal data from open-access sources such as Our World in Data (Global Change Data Lab, n.d.), the World Health Organization's Global Health Observatory (World Health Organization, n.d.a), the Global Observatory on Health Research and Development (World Health Organization, n.d.b), the World Bank Open Data (The World Bank, n.d.a), and similar repositories.

Given that scientific production can be measured, and that socioeconomic indicators are available for the majority of countries across extended time periods, it becomes theoretically possible to estimate, rather than merely speculate about, the principal drivers and barriers of scientific production. In this context, the same analytical framework capable of estimating the alignment of global research with population outcomes (that is, the concept of scientific coherence) can be reformulated by treating global research not as the independent variable, but as the dependent one. This reformulation gives rise to the concept of development coherence, under which it is expected that higher levels of development are associated with a greater capacity to produce scientific research.

## The DAIL framework

**Figure 1:**
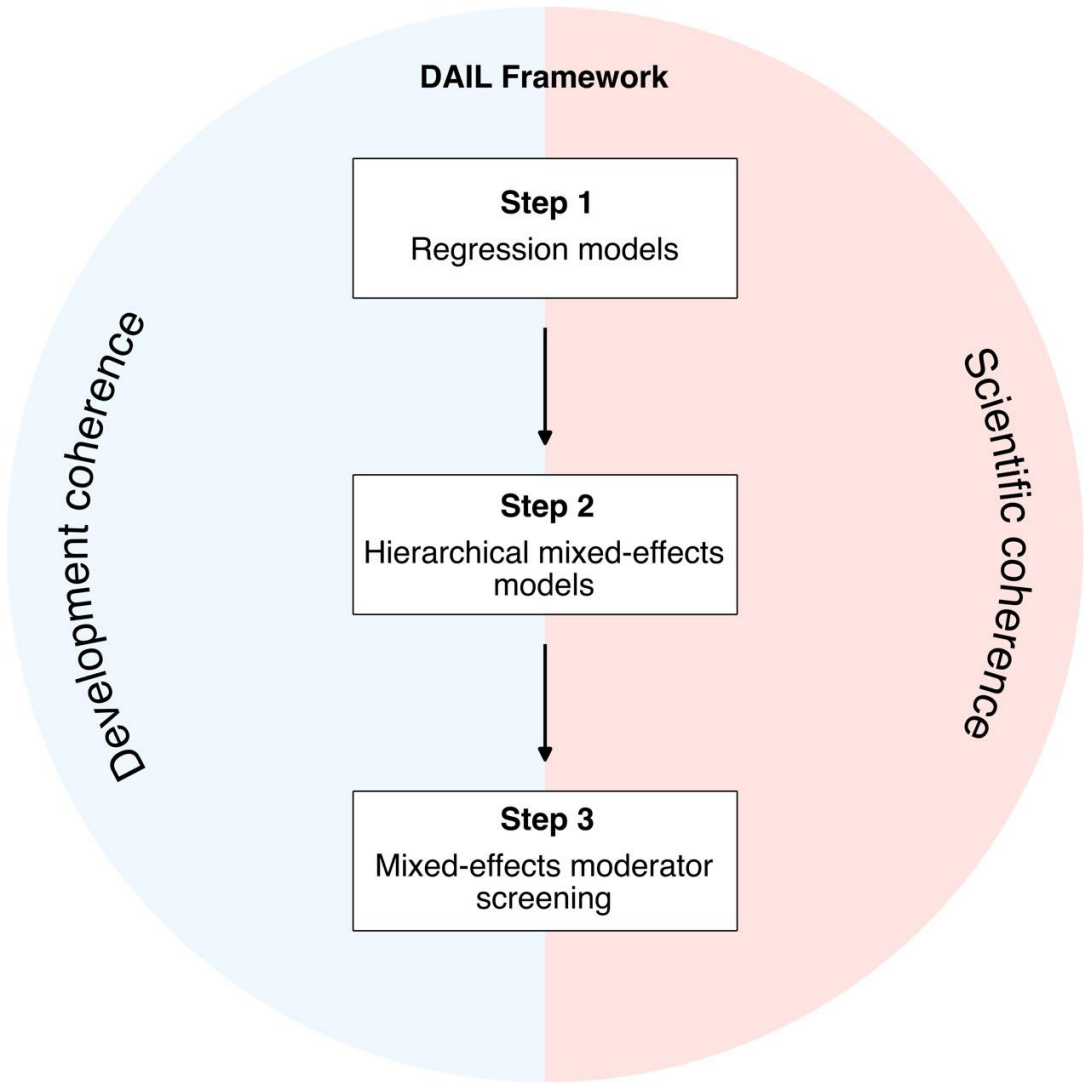
Conceptual overview of the DAIL framework. Step 1 estimates bivariate associations using regression models; step 2 pools evidence across strata using hierarchical mixed-effects models; and step 3 evaluates contextual heterogeneity through mixed-effects moderator screening, jointly informing scientific and development coherence.

Once we have highlighted the knowledge gap, and what kind of methodology is relevant and necessary nowadays, in this section we will propose a solution to precisely fill this gap: the Data-driven Analysis and Inference of Longitudinal population indicators and research production (DAIL) framework, which is a three-step analytical pipeline combining population-level regression models (step 1), hierarchical mixed-effects models (step 2), and mixed-effects moderator screening using cluster-restricted moderators (step 3), to estimate scientific and development coherence across geographical distributions such as countries, income groups, regions, or other strata (Figure 1).

To perform an estimation of scientific coherence using the DAIL framework, three basic components become necessary: (1) bibliometric data, (2) population-level indicator data, and (3) an analytical pipeline to estimate coherence using the latter two components. Each of these three components composes the pillars of coherence estimation in the DAIL framework and will be explained in the following sections. Therefore, this work serves not only as a methodological proposal but also as a practical user guide.

## Data sources

### Bibliometric data

This methodology assumes that the user has access to a structured dataset in which bibliometric records can be linked to population-level indicators. In practice, this begins with a systematic search of major bibliographic databases to retrieve the full body of literature relevant to the research domain of interest. After records are exported and curated, the dataset must include, at minimum, the publication year and author affiliation information of each article. These fields enable the inference of country-level metadata required for alignment with contextual indicators. When multiple affiliations are present, a pragmatic and commonly used approach is to treat the first listed affiliation of the first author as a proxy for the article's country of origin.

### Population-level indicators data

Once country and publication year have been inferred for each record, the user will typically observe repeated country-year combinations because each row corresponds to a single article. To align research production with population-level indicators, the publication-level dataset should therefore be collapsed to a country-year panel by computing the total number of publications per country and year (and, optionally, additional bibliometric summaries such as total citations or journal-quartile counts). This country-year bibliometric panel constitutes the base structure for merging with population-level indicators, which are usually reported at the same country-year resolution.

Indicator selection is necessarily context dependent and does not follow a single mandatory list; however, indicators should be chosen *a priori* and justified based on their conceptual relevance to the domain under study and their plausibility as outcomes hypothesized to co-evolve with the research area (for scientific coherence analyses) or as structural determinants of research production (for development coherence analyses).

### Aggregation to macro-geographic strata

While the DAIL framework is inherently flexible and can be directly implemented at the granular country-year level using nested hierarchical models, for interpretability and comparative reporting, it is often useful to further aggregate country-year panels into macro-geographic strata, such as World Bank income groups (The World Bank, n.d.b) or World Health Organization regions (World Health Organization, n.d.c). Because contextual indicators are defined at the country level, aggregation should preserve population structure. A reproducible approach is to compute, for each stratum and year, population-weighted means of each indicator across all countries in the stratum, using country population in that year as weights, and to retain the total stratum-year population by summing country populations.

In parallel, bibliometric summaries should be computed at the same stratum-year level. The population-weighted indicator panel is then merged with the bibliometric panel by stratum and year, yielding stratum-year datasets that support downstream analyses while remaining directly traceable to the underlying country-year data. This modularity ensures that researchers can choose between macro-aggregated approaches or unaggregated country-level evaluations depending on their specific comparative objectives.

## Analytical steps

### The first step: regression models

In the first step, the aim of the framework is to estimate the associations between bibliometric and population-level data using bivariate regression models. It is important to highlight that the nature of the regression model determines whether we are studying scientific or development coherence. In the former, the dependent variable is the indicator, whereas in the latter, the indicator assumes the role of the independent variable. It is also important to note that, while the number of publications is always a numerical variable with whole numbers, this is not the case for all indicators, as some may be expressed as percentages, ratios, or raw counts with a large magnitude (e.g., the indicator *Deaths per year* from Our World in Data).

Therefore, the framework applies different types of regression models according to the nature and distribution of the variables using a standardized decision rule. Specifically, dependent variables representing whole-number counts are modeled using Poisson regression, or Negative Binomial regression if the data exhibits significant overdispersion. Variables bounded between 0 and 1, such as proportions, are modeled using Quasi-binomial regression. Continuous, strictly positive variables with strong right-skewness are evaluated using Log-linear models, while all other numeric indicators default to standard Linear models. As a result, the regression coefficient is not always a β, but may also be an odds ratio (OR) or an incidence rate ratio (IRR); however, the framework selects a single model for each association based on the pooled data to ensure comparability across macro-geographic locations.

The DAIL framework is designed to estimate scientific or development coherence across a wide range of variables. For this reason, (1) analyses are usually restricted to a defined time window; (2) models are fitted only when there is sufficient information to support them (i.e., avoiding strata-indicator combinations with too few usable observations); (3) robust standard errors are utilized to account for the temporal structure of the longitudinal data; and (4) because the framework may involve testing a large number of associations in parallel, the reported *p*-values are adjusted using the Holm method, thereby reducing the rate of false-positive results.

**Figure 2:**
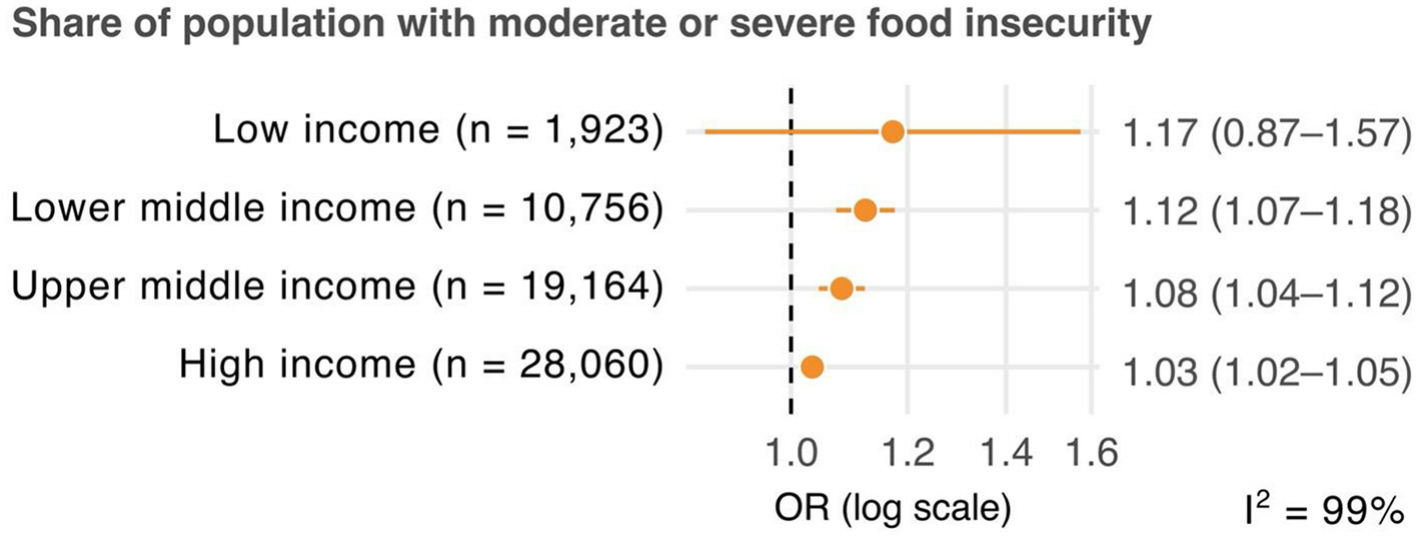
Step 1 regression estimates for the association between scientific production (per 1,000 food-security publications) and the share of population with moderate or severe food insecurity, stratified by World Bank income group.

Maybe this will be easier to demonstrate using an example of how the framework operates with real data. Let us retrieve, as an example, theoretically all published articles on food security using the systematic search strategy described in Supplementary Material 1, which comprises 61,158 peer-reviewed journal articles after the filtering steps outlined in the same supplementary material. For this occasion, we use the indicator *Share of the population with moderate or severe food insecurity* from Our World in Data. When applying this model to explore scientific coherence, we estimate that every 1,000 additional published articles are associated with a 3.3% increase in the odds of a higher share of this population in high-income countries, an 8.2% increase in upper-middle-income countries, and a 12.3% increase in lower-middle-income countries, with no conclusive results observed in low-income countries (Figure 2).

Based on these data-driven estimations of scientific coherence, a discussion could be formulated around the idea that research on food security may not be as effective as expected. However, the models in this step rely on a very optimistic assumption: that the interaction between scientific production and the food-security indicator in this case is the only relevant one, as no other variables are included in the model. Therefore, the next steps will aim to address this limitation, acknowledging that additional variables are likely to influence this outcome.

### The second step: hierarchical mixed-effects models

Modeling regions and income groups separately may not be enough to fully capture the global relevance of the research, either in terms of its scientific coherence or its development coherence. This gap motivated the addition of a second step.

In the second step, DAIL moves from stratum-specific regressions to a hierarchical panel-modeling framework that leverages the repeated-measures structure of the aggregated data. For each indicator-exposure pairing, we fit a mixed-effects model to stratum-year observations, treating strata (e.g., income groups or regions) as the clustering unit and years as the temporal index. The main aim of this step is to establish the foundation for the third step. It is common that, when aggregating results across macro-geographic strata, non-significant coefficients are obtained because the variability between, for example, different income groups or regions is so large that the 95% confidence interval crosses the point of no association.

Occasionally, some associations may remain significant, which can reinforce a potential relationship and should therefore be reported; however, null findings are common and, precisely for this reason, warrant further investigation to identify the main sources of heterogeneity, an objective that underpins the third step.

In this step, models include the focal predictor and a year term to account for secular trends, with a random intercept to capture between-stratum differences. When supported by the data, a random slope for the focal predictor is also included to allow associations to vary across strata. Model families are selected according to the outcome scale to ensure interpretability, and predictors may be rescaled when required for numerical stability. The parameter of interest is the fixed effect of the focal predictor, reported in interpretable units consistent with the link function and accompanied by confidence intervals and *p*-values. Because multiple models are fitted in parallel, statistical inference accounts for multiplicity through appropriate *p*-value adjustments.

### The third step: mixed-effects moderator screening using cluster-restricted moderators

In the third step, DAIL evaluates whether the association identified in Steps 1–2 varies systematically across contextual conditions. That is, this step aims to estimate the magnitude of the effect of third variables, referred to as moderators, on the base model.

To estimate these moderating relationships, DAIL performs a targeted moderation screening in which only a predefined, theory-driven set of potential moderators is evaluated. For each association between research production and an indicator, DAIL fits a mixed-effects model that includes the main predictor, the moderator, their interaction, a time term to account for secular trends, and a random effect to capture differences between strata over time. To minimize structural collinearity, continuous predictors and moderators are mean-centered prior to modeling, and each moderator is evaluated independently (one-at-a-time). Furthermore, models are only fitted when supported by adequate statistical power, enforced by a minimum threshold of 25 usable observations. The goal is to assess whether the strength or direction of the association varies systematically across contextual conditions. To aid interpretation, moderation patterns are summarized using conditional estimates at representative levels of the moderator (e.g., lower vs. higher levels).

Because many moderators are tested in parallel, this step is treated as exploratory. Statistical evidence is therefore adjusted for multiple testing using the False Discovery Rate (Benjamini–Hochberg) method across all evaluated interactions, and the results are intended to highlight a small, interpretable and significant set of moderators.

For example, in the first step we estimated that research on food security may not co-evolve with reductions in the *Share of the population with moderate or severe food insecurity* (Figure 2). However, when incorporating the *State Capacity Index* as a moderator, the association between research output and the corresponding indicator was modified. Specifically, each additional 1,000 publications were associated with a potentially 8.5% smaller change in the odds per unit increase in the State Capacity Index (*OR* = 0.915; 95% *CI*: 0.85–0.98; *p* = 0.046). At lower state capacity (25th percentile), 1,000 additional publications were associated with a 13% increase in the odds (*OR* = 1.139; 95% *CI*: 1.06–1.21), compared with a 6.9% increase at higher capacity (75th percentile; *OR* = 1.069; 95% *CI*: 1.03–1.10).

Thus, in settings with higher state capacity, the association of scientific production may be more favorable and potentially inversely associated with moderate or severe food insecurity. This approach can be applied using the predefined, theory-driven set of moderators, in order to identify the main drivers or barriers influencing the potential translation (i.e., alignment) of research within its corresponding field.

## Discussion

### Evolution of the framework

In this paper, we have presented a novel framework to estimate scientific and development coherence, concepts that have already been defined in this work. The development of this method has followed a continuous, step-by-step process that has evolved over time. In accordance with the philosopher Karl R. Popper, every genuine test of a theory is an attempt to falsify it, or refute it (Popper, 1963). Thus, this is not the first iteration of the method. Rather, it has been progressively refined, with each new version explicitly revealing the potential false-positive rates of the previous ones. From this perspective, a researcher should not aim to demonstrate why a finding may be true, but rather why it may be false (Popper, 1959). Consequently, as we are still attempting to identify the main fallacies of the present version of the method, we consider it appropriate to explain why previous versions were more likely to be false, biased or less informative.

The earliest version consisted of a simple correlation analysis between scientific production, measured using a systematic search strategy, and selected global health, research, and development indicators (Angarita-Pacheco et al., 2025). The magnitude of a correlation coefficient, whether Pearson or Spearman, ranges from −1 to 1. For example, in this context, a correlation of 1 would indicate that as scientific production on medical errors (the example used in the cited paper) increases, the related indicator follows a similar pattern. However, both variables could increase or decrease simultaneously, and such an analysis does not provide an interpretable estimate of how much scientific production is required to generate a benefit, or a negative effect, on the indicator. In other words, a meaningful and interpretable measure of association cannot be obtained using this methodology.

The logical next step was therefore the use of regression models, which address this limitation. This approach was applied in a paper that, although published before the correlation-based study, was analytically developed after identifying regression modeling as the appropriate progression (Lozada-Martinez et al., 2025). Regression models allowed us to estimate more interpretable magnitudes of association for increases in scientific production relative to indicators related to the research domain under study. At this stage, the early version of the DAIL framework consisted of a single step: regression models stratified by macro-geographic location, combined with a systematic search strategy.

However, it soon became evident that relying on separate models by region or income group was insufficient to answer the broader, global question of how useful the research under study actually is (in terms of scientific coherence), or what its main drivers and barriers are (in terms of development coherence). This motivated the introduction of a second step: a meta-analysis of the regression results from Step 1. Some studies using this approach have since been published (Revolledo Caicedo et al., 2025; Romero-Freile et al., 2025; Hernández-Paez et al., 2026a).

Despite these advances, important gaps remained. First, the approach relied on the optimistic assumption that linear regression models provided the best fit for all estimated associations, an issue that is explicitly addressed in the current version of Step 1. Second, because the initial regression models were biased by this assumption, the subsequent meta-analyses were also biased, leading to what could be described as bias compounded upon bias. As a result, this approach was not ideal for producing a robust global assessment, as it depended heavily on potentially misspecified estimates and standard errors. This limitation motivated the transition to hierarchical mixed-effects models, which offer a stronger and more flexible analytical framework.

Finally, earlier attempts to assess the moderating role of third variables relied on meta-regression applied to meta-analytic results, which, to our understanding, introduced an additional layer of bias, given that the meta-analyses themselves were already affected by the exclusive use of linear regression models. Therefore, in the current version of the framework, meta-regression was replaced by a mixed-effects moderator screening using cluster-restricted moderators, which fulfills the same purpose. Studies utilizing this updated methodological pipeline, integrating both hierarchical mixed-effects models and moderator screening, have recently been published (Sarquis Rivera et al., 2026; Acosta-Monterrosa et al., 2026a; Hernández-Paez et al., 2026b).

### Implementation science and knowledge translation

Within the field of implementation science, broad dissemination is recognized as a foundational principle. The scientific method relies on the premise that findings must be accessible to be discussed, challenged, and reproduced; consequently, the cumulative advancement of knowledge operates optimally only when research is openly available to the global scientific community (Schiltz, 2018). Furthermore, the diffusion of scientific knowledge is a complex social process governed by multiple determinants extending well beyond the empirical strength of the innovation itself (Bauer and Kirchner, 2020). The urgency for methodological frameworks to study this diffusion is underscored by the persistent implementation gap (the time required for scientific discoveries to reach widespread practice), which currently averages 17–20 years (Bauer and Kirchner, 2020; de Waard et al., 2025; Mosteller, 1981).

In alignment with the core objectives of implementation science, the DAIL framework aims to identify and estimate the primary barriers and facilitators that modulate the association between research production and population outcomes. Building on this foundation, future iterations of the model should explicitly account for this temporal latency to adjust and design lag-aware longitudinal analyses. Understanding these variables is crucial for developing targeted strategies that overcome systemic barriers, leverage facilitators, and ultimately accelerate the translation of research into tangible societal value.

### Publication metrics, accessibility, and systemic bias

This imperative for accessible science raises critical considerations for the application of our framework. Foremost, the intrinsic quality of research should be evaluated by its substantive content rather than superficial proxy metrics, such as journal impact factors or quartiles (Zhang et al., 2017; Acosta-Monterrosa et al., 2026b). Traditional metrics heavily influence an article's visibility and citation rate, introducing significant bias. For example, high-quality research originating from the Global South often faces systemic barriers, such as publication paywalls, that impede its widespread dissemination and subsequent citation (Moutinho, 2024). Because citation counts are heavily confounded by these access disparities, they may represent a biased indicator; thus, they were intentionally excluded from our current proof-of-concept example. Nevertheless, in certain contexts, incorporating these metrics could serve as a valid adjustment for the models.

Additionally, assigning research output based on the first author's affiliation does not account for cross-border research dynamics (such as authors from one country analyzing data from another), nor does it capture the complex economic externalities of publishing, such as the potential drain of research resources through Article Processing Charges (APCs) paid to international publishers. To mitigate these limitations, future applications of the framework could employ fractional authorship counting methods to more accurately capture collaborative cross-border contributions, while integrating country-level open-access expenditure data as explicit structural covariates.

### The challenge of scientific fraud and paper mills

A primary concern regarding the framework's heavy reliance on publication volume is the potential bias introduced by the artificial inflation of research production driven by scientific fraud or “paper mills,” the process by which manufactured manuscripts are submitted to journals for a fee to secure publication on behalf of researchers or to offer authorship for sale (Mainous, 2025). While these phenomena remain challenging to systematically identify and exclude at a macro-level, this bias may currently be relatively minor in the context of the framework, as the estimated proportion of global literature originating from paper mills ranges between 1.5% and 5% (Parker et al., 2024; Van Noorden, 2023). For context, a recent landscape analysis of biomedical literature evaluated 20,687,150 papers with valid English abstracts indexed in PubMed; the study identified 11,756 retracted papers, observing that they predominantly clustered in specific research areas known to be major targets for paper mills (González-Márquez et al., 2024).

Although the lack of a standardized, definitive method means some manufactured papers will inevitably evade current detection protocols, robust countermeasures are actively being developed. These include automated identification and discovery tools designed by third-party organizations such as Clarivate (Parker et al., 2024) and researchers like Byrne and Labbé (Labbé et al., 2019). Furthermore, large language models trained on PubMed datasets have demonstrated utility in flagging fraudulent publications (González-Márquez et al., 2024) by identifying textual patterns and structural similarities that raise suspicion (Parker et al., 2022; Christopher, 2021). Consequently, researchers utilizing the DAIL framework pipeline should consider applying these computational filtering tools to exclude potential paper-mill products from their bibliometric datasets prior to executing the three-step analysis.

### Analytical limitations of the DAIL framework

Several other limitations must be acknowledged in the use of this methodology. First, the framework relies on aggregated population-level data, which precludes causal inference and may be affected by ecological bias. Second, research production is measured using bibliometric proxies based on raw publication counts, which do not fully capture research quality, implementation, or translational pathways. Third, temporal associations between scientific activity and population indicators may be confounded or modified by translation lag effects, structural confounding, implementation capacity, and measurement variability across countries and data sources (Samet and Ness, 2012).

### Proof-of-concept and future directions

As a methodological proof-of-concept, the empirical examples presented here are intended to illustrate the analytical approach of the DAIL framework rather than provide definitive estimates of scientific or development coherence within the specific domain of food security. Consequently, an in-depth discussion of specific domain results falls outside the scope of this paper. As detailed in our methodological evolution, earlier iterations of this analytical approach have been empirically tested across diverse research fields (Lozada-Martinez et al., 2025; Angarita-Pacheco et al., 2025; Revolledo Caicedo et al., 2025; Romero-Freile et al., 2025; Hernández-Paez et al., 2026a). However, the current manuscript does not present a final or definitive version of the framework. While earlier versions have successfully undergone peer review, this does not imply methodological infallibility; rather, it subjects the framework to necessary academic scrutiny.

The primary aim of this study is to formalize the current iteration of the DAIL framework, demonstrate its analytical potential, and explicitly outline the existing methodological gaps that remain to be filled. We acknowledge that the development of this framework is an iterative process. Future methodological iterations should explore incorporating citation-weighted, field-normalized, or quality-adjusted measures to account for the heterogeneous contributions of different research outputs. Ultimately, we present this framework to the multidisciplinary scientific community so that future applied research can leverage, critique, and refine this pipeline to conduct deep, domain-specific investigations into the complex dynamics between research production and population-level trajectories.

## Conclusion

We have proposed a method to estimate two novel concepts introduced in this work: scientific and development coherence. Throughout the paper, we have shown that results obtained using earlier versions of this framework have been published in peer-reviewed, high-impact journals, demonstrating both the conceptual plausibility and methodological utility of the approach and its relevance when results are properly conceptualized and interpreted. These earlier applications have also shown the framework's ability to address important meta-scientific questions. Such questions can be formulated and addressed using the DAIL framework across any research domain worldwide. However, the proxy nature of these estimations should be emphasized, as the framework is not designed to establish causality but rather to quantify potential associations within the context of scientific and development coherence.

## Data Availability

The original contributions presented in the study are included in the article/Supplementary material, further inquiries can be directed to the corresponding author.
