# Supplementary material for: Estimating scientific coherence using population-level indicators and research production data: a longitudinal analytical proof-of-concept study
Source: Front Res Metr Anal. 2026 Jun 29;11:1817595. doi: 10.3389/frma.2026.1817595 (PMC13357400; doi:10.3389/frma.2026.1817595)
Supplement: Supplementary file 1 [file Data_Sheet_1.docx]

**Supplementary Material 1. Systematic search strategy for food-security scientific production.**

**Data source and search date**

Scientific research output on food security was identified through a comprehensive search of the Scopus database. The search was conducted on 5 November 2025 using title and abstract fields.

**Search strategy**

The following query was applied in Scopus:

TITLE-ABS(“Food Security”) OR TITLE-ABS(“Access to Healthy Food*”) OR TITLE-ABS(“Healthy Foods Availabilit*”) OR TITLE-ABS(“Access to Health Food*”) OR TITLE-ABS(“Healthy Food Availabilit*”).

This search was constructed using the MeSH Unique ID: D000082302, and MeSH Unique ID: D000091483.

**Record retrieval and eligibility filtering**

The initial search retrieved 80,309 records. Document types not representing original, peer-reviewed research were excluded, including book chapters (n = 9,351), conference papers (n = 6,599), books (n = 1,167), errata (n = 210), conference reviews (n = 143), and retracted publications (n = 41), resulting in 62,798 records. After excluding articles in press (n = 925) and additional non-article sources (book series, trade journals, books, and conference proceedings; n = 715), a final dataset of 61,158 peer-reviewed journal articles was retained and included in the analysis.

**Metadata extraction and construction of analytic panels**

For each publication, metadata on publication year and country affiliation were extracted. Publications were assigned to countries based on corresponding author affiliation and subsequently aggregated by World Bank income group and year to construct annual measures of food security research output.
